# HBB contributes to individualized aconitine-induced cardiotoxicity in mice via interfering with ABHD5/AMPK/HDAC4 axis

**DOI:** 10.1038/s41401-023-01206-3

**Published:** 2024-03-11

**Authors:** Ya-juan Guo, Jing-jing Yao, Zhen-zhen Guo, Ming Ding, Kun-lin Zhang, Qing-hong Shen, Yu Li, Shao-fang Yu, Ting Wan, Fu-ping Xu, Ying Wang, Xiao-xiao Qi, Jin-jun Wu, Jian-xin Chen, Zhong-qiu Liu, Lin-lin Lu

**Affiliations:** 1 Joint Laboratory for Translational Cancer Research of Chinese Medicine of the Ministry of Education of the People’s Republic of China, Guangdong-Hong Kong-Macau Joint Lab on Chinese Medicine and Immune Disease Research, International Institute for Translational Chinese Medicine, Guangzhou University of Chinese Medicine, Guangzhou, 510006 China; 2 State Key Laboratory of Quality Research in Chinese Medicine, Macau University of Science and Technology, Macau, China; 3 Guandong Provincial hospital of Chinese Medicine, Guangzhou University of Chinese Medicine, Guangzhou, Guangdong 510006 China; 4 Beijing University of Chinese Medicine, Beijing, 100029 China

**Keywords:** aconitine, cardiotoxicity, individual differences, hemoglobin subunit beta, nitrogen monoxide, ABHD5/AMPK/HDAC4 axis

## Abstract

The root of *Aconitum carmichaelii Debx*. (Fuzi) is an herbal medicine used in China that exerts significant efficacy in rescuing patients from severe diseases. A key toxic compound in Fuzi, aconitine (AC), could trigger unpredictable cardiotoxicities with high-individualization, thus hinders safe application of Fuzi. In this study we investigated the individual differences of AC-induced cardiotoxicities, the biomarkers and underlying mechanisms. Diversity Outbred (DO) mice were used as a genetically heterogeneous model for mimicking individualization clinically. The mice were orally administered AC (0.3, 0.6, 0.9 mg· kg^−1^ ·d^−1^) for 7 d. We found that AC-triggered cardiotoxicities in DO mice shared similar characteristics to those observed in clinic patients. Most importantly, significant individual differences were found in DO mice (variation coefficients: 34.08%–53.17%). RNA-sequencing in AC-tolerant and AC-sensitive mice revealed that hemoglobin subunit beta (HBB), a toxic-responsive protein in blood with 89% homology to human, was specifically enriched in AC-sensitive mice. Moreover, we found that HBB overexpression could significantly exacerbate AC-induced cardiotoxicity while HBB knockdown markedly attenuated cell death of cardiomyocytes. We revealed that AC could trigger hemolysis, and specifically bind to HBB in cell-free hemoglobin (cf-Hb), which could excessively promote NO scavenge and decrease cardioprotective S-nitrosylation. Meanwhile, AC bound to HBB enhanced the binding of HBB to ABHD5 and AMPK, which correspondingly decreased HDAC-NT generation and led to cardiomyocytes death. This study not only demonstrates HBB achievement a novel target of AC in blood, but provides the first clue for HBB as a novel biomarker in determining the individual differences of Fuzi-triggered cardiotoxicity.

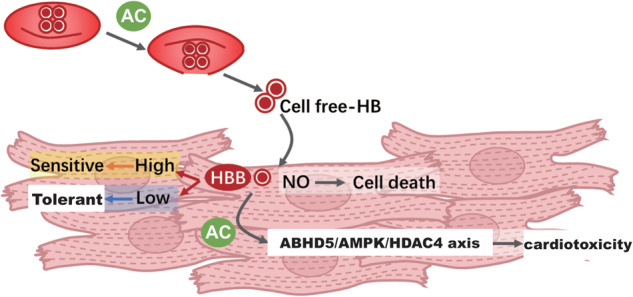

## Introduction

The root of *Aconitum carmichaelii Debx*. (Fuzi) is a classic herbal medicine used in China for thousands of years to treat heart failure [1], pulmonary hypertension [2], kidney failure [3], rheumatologic [4, 5], and hematologic diseases [6]. Given its predominant and irreplaceable efficacy in rescuing patients from severe and fatal diseases, Fuzi is also recognized as “General”, governing other herbal medicines. Unfortunately, the clinical application of Fuzi has been seriously hindered. The greatest obstacle is the unpredictable and severe toxicity triggered by Fuzi, and these effects are accompanied by its strong effectiveness. The toxicities of Fuzi are gastrointestinal toxicity, including nausea, vomiting, diarrhea, and respiratory toxicity, including respiratory depression [7]. However, the most fatal toxicities are arrhythmia and ventricular fibrillation, which account for ~5.5% of sudden deaths in Fuzi-intake patients [8]. Therefore, a comprehensive understanding of the characteristics of Fuzi-triggered cardiotoxicities in the clinic and the underlying molecular mechanisms are urgently needed.

Numerous clinical Fuzi poisoning cases have demonstrated that a high individualized toxicity response is the predominant and principal feature of Fuzi-associated cardiotoxicities. Previous studies revealed that ~88.2% of patients may suffer from individualized mild to moderate arrhythmias within 2 h after Fuzi intake [9]. In the Chinese pharmacopoeia, the recommended dosage of Fuzi is 3–15 g, while in ~51.02% of clinical prescriptions, the dosages of Fuzi were actually far beyond this recommended dose [10]. Especially for certain patients who might tolerate Fuzi, to gain more effectiveness, the amount of Fuzi could reach as high as 500 g, and strikingly, no severe toxicities were observed in these Fuzi-tolerant patients [11]. Similar to digoxin and warfarin, Fuzi also demonstrates a rapid transition from onset to poisoning; even worse, the toxic dosage of Fuzi is unpredictable due to individualized response sensitivities [9]. Thus, high individual response variation and the triggering of unexpected and inevitable Fuzi-related cardiotoxicities are the greatest obstacles hindering the clinical safety applications of Fuzi. However, although overwhelming evidence has demonstrated the molecular mechanism of Fuzi-related toxicity, few studies have focused on the individual differences and biomarkers involved.

Aconitine (AC), the principal toxic component in Fuzi, can induce fatal arrhythmia and heart arrest at a minimum dosage of 0.2 mg and cause sudden death at 1–2 mg [12]. The main mechanisms underlying AC-triggered cardiotoxicity are as follows: *(i) interference with ionic homeostasis*: modulating Na^+^/K^+^/Ca^2+^ ion efflux/influx, disrupting ionic homeostasis in cardiomyocytes, and triggering arrhythmias [7]; *(ii) promotion of myocardial injury*: increasing [Ca^2+^]_i_, reducing mitochondrial membrane potential (ΔΨm), and activating mitochondria-mediated and autophagy-related apoptotic pathways to promote cardiomyocyte death [7]; and *(iii) regulation of redox homeostasis*: imbalance of oxidation/reduction triggers cell membrane lipid peroxidation, leading to cardiomyocyte necrosis and local vasoconstriction to aggravate arrhythmias. Unfortunately, although in AC-related cardiotoxicity, some molecular targets have been identified, such as Na^+^/K^+^ ATPase (NKA), Na^+^ /Ca^2+^ exchanger (NCX), and L-type Ca^2+^ channel, the combination of their corresponding inhibitors/agonists with AC still cannot effectively attenuate AC-triggered cardiotoxicity [13]. Thus, novel toxic biomarkers that could specifically define the individual responsive sensitivity to AC-associated cardiotoxicity are urgently needed.

DO (diversity outbred) mice were developed by randomly breeding eight strains of inbred mice, including A/J, C57BL/6J, 129S1/SvImJ, NOD/ShiLtJ, NZO/HlLtJ, CAST/EiJ, PWK/PhJ, and WSB/EiJ [14]. Given its highly heterozygous genetic background (~38 million SNPs and 7 million variants), the DO mouse is an ideal and classic animal model for individualized research, especially to mimic the individual response of patients to drug treatments [15]. Therefore, by utilizing a DO mouse model, our study will provide the first evidence to reveal the individual differences in AC-induced cardiotoxicities and the underlying biomarkers and mechanisms.

## Materials and methods

### Chemicals and reagents

Aconitine (AC, MUST-18110905; CAS ID: 302-27-2), Aconine (CAS ID: 509-20-6), and Benzoylaconine (BAC, MUST-19060306; CAS ID: 466-24-0) (purity >98%) were purchased from Chengdu Man site Pharmaceutical Co., Ltd. (Chengdu, China). DMEM and fetal bovine serum (FBS) were purchased from Gibco (NY, USA). Primary antibodies against β-actin (sc-8432), IgG-HRP (sc-2004 and sc-2005) were obtained from Santa Cruz Biotechnology, Inc (CA, USA). Antibodies against HBB (ab214049), ADH1 (ab108203), ABHD5 (ab59488), S-Nitrosylation (ab236207), eNOS (ab199956), iNOS (ab283668), nNOS (ab5583), and GAPDH (ab181602) were obtained from Abcam (Cambridge, UK). Antibodies against AMPKα (5832), p-AMPKα (2535), mTOR (2983), p-mTOR (2974), and HDAC4 (7628) were obtained from Cell Signaling Technology (Boston, MA, USA).

### Animals

All animal experiments were approved by the Animal Experimental Committee of Guangzhou University of Chinese Medicine (Guangdong, China) and carried out in accordance with the experimental animal management of Guangdong province. DO mice and eight strains of DO progenitor mice (A/J, C57BL/6J, 129S1/SvImJ, NOD/ShiLtJ, NZO/ HlLtJ, CAST/EiJ, PWK/PhJ, and WSB/EiJ) were supplied by the Jackson Lab. All mice were about 6–7 weeks old and weighed 16–18 g. After quarantine, the mice were maintained in a Specific Pathogen Free animal laboratory (License number: SYXK (GZ) 2019-0144) with 12 h light/dark cycle.

### Individual differences of AC-triggered cardiotoxicity in DO and progenitor mice

188 DO mice were randomly divided into three groups: No. 1–63 treated with AC (0.3 mg ·kg^−1^ ·d^−1^, *n* = 63), No. 64–126 treated with AC (0.6 mg ·kg^−1^ ·d^−1^, *n* = 63), and No. 127–188 treated with AC (0.9 mg ·kg^−1^ ·d^−1^, *n* = 62). AC was administered intragastrically (*per os, po*) for continuously 7 d. The toxicity symptoms were evaluated on the d 1, d 3, and d 5, and scored every 5 min within 360 min after administration according to scoring criteria shown in Table 1. On the d 1 and d 2 of AC exposure, ECG, animal behavior, and blood were simultaneously collected to evaluate the initial or the ultimate response for Fuzi-associated cardiotoxicity. Subsequently, the mice were sacrificed by cervical dislocation, the hearts were rapidly removed and stored at −80 °C or fixed with 4% paraformaldehyde. Blood samples were centrifuged at 4800 *×g* for 10 min at 4 °C, and the plasma was collected and stored at −80 °C. Mice with high toxicity scores (score > 15) and abnormal ECGs were considered as AC-sensitive mice. Mice with low toxicity scores (score < 8) and normal ECGs are recognized as AC-tolerant mice. Mice with a slightly abnormal ECG and toxicity scores in the range of 8–15 were defined as a medium type.

**Table 1:** AC toxicity symptom scoring criteria.

Scores	0	1	2	3	4
Evaluation method					
Respiratory system	The mice breathed normally and their abdomen fluctuated evenly	The mice had secretions in their nostrils and shortness of breath	The mice breathed slowly, abdominal breathing	Breathe deeply	Respiratory paralysis, asphyxial shock
Autonomic nervous system	There were no drowsiness or mania in mice	The mice ran back and forth, jumped, fretted, and somersaulted	The mice were stiff, active and manic	The mice’s limb weakness and body tremor	The mice were paralyzed and could not walk, but the body reacted under the action of external force
Gland secretion	There were no obvious secretions at the corners of the mouse mouth	Retch	Transparent saliva at the corners of the mouth	A lot of vomit came out the corners of the mouse mouth	Keep vomiting
Central nervous and motor nerves	Pinch the tail, the mice quickly turns back, the response is agile, and the movement was flexible	Pinch the tail, the mice could turn back, but with reduced responsiveness and the inflexible body	Pinch the tail, the mice responded, head and body twisted, but were unable to turn back	Pinch the tail, the mice responded with head and body, but were unable to twist	Pinch the tail, the mice didn’t respond

### Survival ratio of progenitor mice

Eight progenitor mice (6–8 weeks, ten number of each ancestry strain, *n* = 10) were selected for intragastric administration of AC for 10 d. The administered dose gradually increased with time. The dose of AC was 0.45 mg· kg^−1^ ·d^−1^ on d 1–3, 0.9 mg· kg^−1^ ·d^−1^ by gavage on d 4 and 5, 1.8 mg ·kg^−1^ ·d^−1^ on d 6–8, and 3.6 mg ·kg^−1^ ·d^−1^ by gavage on d 9 and d 10. The death time and number were recorded for each strain of mice, and mice survival curves were plotted.

### Individual differences in toxicity of AC in NOD/ShiLtJ and 129S1/SvImJ mice

Thirty NOD/ShiLtJ and 129S1/SvImJ mice were given AC 0.3 mg/kg and 0.45 mg/kg by gavage at once, respectively. The toxicity symptoms were scored according to the scoring criteria. ECG was recorded by electrophysiological instrument. The toxicity sensitivity of AC in mice was classified by a combination of ECG and toxicity score. Individual differences in the toxic response of mice to aconitine (AC-sensitive and AC-tolerant mice) were categorized by ECG and toxicity scores.

### Cell lines

Primary heart cells were obtained from the hearts of NOD/ShiLtJ and 129S1/SvImJ mammary rats. The body surfaces of the suckling rats were sprayed with alcohol, placed on an ultra-clean bench, and then the hearts were removed and placed in cooled PBS. Next, the blood was squeezed out of the heart tissue with forceps and then washed with fresh cooled PBS. Next, the heart tissue is cut into uniform pieces. After cutting, the blood in the heart tissue is washed with PBS. Then, the tissue pieces were transferred to conical flasks (ground conical flasks). After the heart tissue was digested with trypsin, it was shaken at 37 °C for 5 min and then the first trypsin digest was discarded. Depending on the number of hearts, a new 5–7 mL of trypsin was added and the tissue was digested at 37 °C for 5 min (shake gently to ensure that the tissue pieces move, but not too vigorously). Then the same volume of 20% DMEM was added to terminate the digestion and the cells were collected in a new centrifuge tube. Next, fresh trypsin was continuously added to the conical flask, it was blowed away with a cut blue tip, and the tissue was continuously digested untile it was lysed. Finally, the collected cells were centrifuged at 500 *×* *g* for 3 min. The supernatant medium was discarded and the cells were resuspended. Then the cells were inoculated. Primary cardiomyocytes were cultured in 10 cm × 10 cm dishes. And AC16 cells were purchased from ATCC (VA, USA). They were cultured in DMEM containing 10% FBS and placed in a cell culture incubator at 37 °C, 5% CO_2_. *Escherichia coli* BL21 and DH5α cell lines were purchased from Tsingke BIO (Guangzhou, China).

### ELISA assay

Creatine kinase isoenzymes (CK-MB) concentrations in the plasma were measured by ELISA kit (Nanjing Jiancheng Bioengineering Institute, Jiangsu, China) according to the manufacturer’s instructions.

### Immunohistochemistry (IHC) staining

Paraffin heart sections of 5 μm were baked at a temperature of 60 °C, then permeabilized with xylene and dehydrated with an ethanol gradient (100%, 90%, 80%, 70%). After continuous incubation with antigen retrieval solution (CWBIO, Beijing, China) and 3% H_2_O_2_ for 30 min, slides were rinsed with water and incubated with anti-HBB, AMPK, ADH1, and ABHD5 antibodies (1:200) at 4 °C overnight. For the negative control, the primary antibody was replaced with unimmunized serum. Next, the slides were rinsed and incubated with the corresponding secondary antibodies (Beijing Biosynthesis Biotechnology Co., Ltd., Beijing, China) for 30 min, followed by 3,3′-diaminobenzidine (DAB) and hematoxylin staining, respectively. The slides were then examined and photographed with an Olympus BX53 fluorescence microscope (Tokyo, Japan). DAB staining was analyzed by Image-Pro Plus 6.0 software.

### Heart hematoxylin-eosin (H&E) and Masson’s trichrome staining

After mice were sacrificed, heart tissue was isolated and fixed in 10% phosphate-buffered formalin for 24–48 h, followed by embedding in paraffin and sectioning (4–5 μm). Dewaxed histological sections were stained with hematoxylin-eosin and Masson’s trichrome.

### Western blot assay

Proteins were extracted from heart tissues or AC16 cells with RIPA lysis buffer (Merck KGaA, Darmstadt, Germany) and quantified by a BCA reagent (Thermo Fisher, MA, USA). HBB proteins were extracted from red blood cells using the Blood Cell Protein Extraction Kit (Beijing Solarbio Science & Technology, Beijing, China). Plasma protein was extracted from plasma using a plasma protein extraction kit (Beijing Baiao Science, Beijing, China). And then quantification is performed by a BCA reagent (Thermo Fisher, MA, USA). Protein lysates were mixed with loading buffer, followed by high temperature (100 °C) denaturation for 5 min. Then, proteins were electrophoretically separated on SDS-PAGE gels and subsequently transferred to PVDF membranes (Pall Life Sciences, NY, USA). Next, PVDF membranes were blocked by 5% BSA for 1 h and hybridized with primary antibodies against HBB, ADH1, ABHD5, AMPKα, p-AMPKα, mTOR, p-mTOR, eNOS, iNOS, nNOS, S-nitrosylation, β-actin and GAPDH. Finally, the membranes were washed with TBST buffer three times for 10 min and analyzed by the Alpha FluorChem E chemiluminescence system (ProteinSimple, CA, USA). The gray values of the bands were quantified by Image J software version 1.4.3.67 (National Institutes of Health, Md, USA).

### siHBB transfection

The HBB siRNA kit was designed and constructed by Guangzhou RiboBio Co., LTD (Guangdong, China), which contains siRNA-NC, siHBB-1, siHBB-2, and siHBB-3. And 1.25 μL siRNA stock solution (20 μM) was diluted into 30 μL riboFECT™ CP Buffer, followed by 3 μL riboFECT™ CP Reagent and incubated at room temperature for 15 min. Primary cardiomyocytes were cultured in 6-well plates grown to 50% confluence and transfected with the HBB siRNA (50 nmol) in Opti-MEM media (Invitrogen) using Lipofectamine™2000 (Invitrogen) according to the manufacturer’s instructions. The sequences of siHBB are as follows: si-m-HBB-bs_001, 5′-GTACTTTGATAGCTTTGGA-3′; si-m-HBB-bs_002, 5′-TGATAACTGCCTTTAACGA-3′; si-m-HBB-bs_003, 5′-TCCTGGGCAATATGATCGT-3′.

### HBB overexpression plasmid transfection

The pEXP-RB-Mam-EGFP-HBB plasmid was designed and constructed by Guangzhou RiboBio Co., LTD (Guangdong, China). AC16 cells were grown to 50% confluence in 6-well plates and transfected with the pEXP-RB-Mam-EGFP-HBB plasmid in Opti-MEM media (Invitrogen) using Lipofectamine™2000 (Invitrogen) according to the manufacturer’s instructions.

### Real-time quantitative polymerase chain reaction (RT-PCR) analysis

mRNA were extracted from primary cardiomyocytes by Trizol reagent (Life Technologies, CA, USA) after cultured with siRNA-NC, siHBB-1, siHBB-2, or siHBB-3 for 12 h. The concentration of RNA was determined by Nano 2000 UV/Vis spectrophotometer (Thermo Fisher, MA, USA). Then, RNA were reverse transcribed into cDNA using Evo M-MLV RT Kit (Accurate Biotechnology, Hunan, China). The HBB expression was assessed by ABI 7500 system (Applied Biosystems, CA, USA) through SYBR Green Premix Pro Taq HS qPCR Kit (Accurate Biotechnology, Hunan, China). The primers are listed as follows: HBB forward:5′-AGGTGAACGCCGATGAAGTT-3′ and reverse: 5′-ATGCAGCTTGTCACAGTGGA-3′; GAPDH forward: 5′-AGGTCGGTGTGAACGGATTTG-3′ and reverse: 5′-TGTAGACCATGTAGTTGAGGTCA-3′. The method of 2^-ΔΔ^^Ct^ was adopted to analyze the data.

### Co-Immunoprecipitation (Co-IP) assay

1 × 10^7^ AC16 cells were lysed with NP40 lysis buffer (1%NP40, 150 mM NaCl, and 50 mM Tris HCl, pH 8.0) and sonicated with a tip ultrasonic homogenizer. Cell lysates were incubated with rabbit IgG (Abcam, Cambridge, UK), anti-HBB antibody (Abcam, Cambridge, UK), and anti-HBB antibody. AC was added and incubated overnight at 4 °C, respectively. Then, the mixture was incubated with mixed dyna-beads A and G (Thermo Fisher, MA, USA) for 2 h at 4 °C. Subsequently, the beads were washed three times with NP40 lysis buffer. And the enriched proteins were eluted and denatured by NP40 lysis buffer supplemented with LDS sample buffer (Thermo Fisher, MA, USA) and DTT (Sangon Biotech, Shanghai, China). The samples were detected by Western blotting and analyzed by the Alpha FluorChem E chemiluminescence system (ProteinSimple, CA, USA).

### Solvent-induced protein precipitation (SIP) assay

This experimental methodology was carried out as previously described [16]. Heart tissues or cells were lysed with NP-40 Lysis Buffer. The cell suspension was freezed with liquid nitrogen and thawed in 37 °C water bath. When the cell suspensions are about 60% thawed, they were placed on ice to continue thawing. This process is repeated three times. The protein lysate was then centrifuged 20,000 *×g* for 10 min at 4 °C, the supernatant was aspirated and divided into the administration group (different concentration gradients could be set) and the solvent group. Drug and protein lysates were incubated for 20 min at room temperature. Denaturation was induced using an organic solvent of acetone/ethanol/acetic acid in a ratio of 50:50:0.1(A.E.A. = acetone: ethanol: acetic acid). The organic solvent is added at a ratio of 9%–19%. The mixture was then equilibrated at 37 °C, 500 *×* *g* for 20 min, then, centrifuged at 20,000 *×* *g* for 10 min at 4 °C and finally the supernatant was collected. A portion of this was used for Western blotting analysis or coomassie brilliant blue staining, and the other portion was stored at −80 °C for subsequent mass spectrometry (MS) quantification.

### Intracellular reactive oxygen species (ROS) detection assay

Primary cardiomyocytes at 2000 cells/well were seeded into 96-well cell culture plates and incubated overnight. Then, the cells were transfected with HBB siRNA or HBB overexpression plasmid for 12 h, followed by 200 μM AC treatment for 24 h. Intracellular ROS was determined by Fluorometric Intracellular ROS Kit (Sigma-Aldrich, MO, USA), according to the instructions. Intracellular green fluorescence intensity was measured by spectrophotometer.

### RNA-seq analysis

RNA-seq analysis was performed on heart tissues from 15 AC-sensitive and 15 AC-tolerant DO mice. Similarly, heart tissues from 6 NOD/ShiLtJ and 6 129S1/SvImJ mice were also sequenced, which contained 3 AC-sensitive or AC-tolerant mice. RNA-seq analysis were supported by the BGI Group. The procedures mainly include total RNA extraction and purification, cDNA synthesis, PCR amplification, library construction, sequencing, and bioinformatics analysis.

### Proteomic analysis

The SIP precipitates were further subjected to proteomic analysis by 4D-LFQ (Jingjie PTM Bio Labs). The main experimental procedures of proteomic analysis mainly included protein extraction, trypsin digestion, LC-MS/MS analysis, and bioinformatics analysis. Each sample was washed with acetone, dispersed by sonication, and digested with trypsin overnight. Subsequently, the peptides were separated and analyzed by EASY-nLC1200 ultra-high performance liquid chromatography system and OrbitrapExploris ™480 MS.

### HBB protein purification

HBB [NM_000518.5:51–494 Homo sapiens hemoglobin subunit beta (HBB), mRNA] pET-28a (+) recombinant plasmid was constructed by Gene Pharma (Shanghai, China), and transferred to *Escherichia coli* (*E.coli*) BL21 (DE3) cells to expand at 37 °C, which restriction enzyme cutting sites were Nde1 and Sal1. Then, protein expression was induced with 1 mM isopropyl b-*D*-1-thiogalactopyranoside (IPTG) for 3–5 h. Next, the E. coli transfected with HBB pET-28a (+) plasmid was broken in buffer A (40 mM Tris-HCl, pH 8.0, 250 mM NaCl, 10 mM imidazole, 1 mM β-Mercaptoethanol and 1 mM protease inhibitor), then centrifuged at 20,000 *×* *g* for 1 h at 4 °C. The target protein was purified by Ni^2+^-Nitrilotriacetic (Ni-NTA) agarose resin and eluted with buffer B (250 mM NaCl, 40 mM Tris-HCl, 250 mM imidazole). The proteins were condensed and stored at −80 °C.

### Experiment of HBB protein replenishment in vivo

12 NOD/ShiLtJ mice were selected and given 0.3 mg/kg AC by gavage on the d 1, to detect the acute toxicity of AC and cardiotoxicity-related behavior scores. Twelve mice were randomly divided into two groups of 6 mice each on the d 2. Then, 125 mg/kg IgG and HBB protein were injected via tail vein, respectively. After 60 min, 0.3 mg/kg AC were administered by gavage. And changes in the cardiotoxicity-related behavior scores of mice were observed on the d 1 and d 2.

### Auto dock analysis

The 3D structure of AC was obtained from PubChem (https://pubchem.ncbi.nlm.nih.gov) and transformed into a pdbqt file format by raccoon software. The crystal structure of HBB (PDB ID: 5KER), ADH1 (PDB ID: 4W6Z), ABHD5 (PDB ID: 5A4H), and AMPK (PDB ID: 2LTU) were obtained from the Protein Data Bank (PDB) database (https://www.rcsb.org/) and converted into a PDBQT format file by MGL Tools 1.5.6 software. After setting the active center, Autodock vina software was used to complete the molecular docking.

### In vitro hemolysis assay

Blood was taken from mice orbits in 1.5 mL tubes containing EDTA, then centrifuged at 500 *×* *g* for 15 min. The supernatant was discarded and the blood cells were resuspended and rinsed three times with alcohol solution. Blood cells were resuspended and washed three times with PBS. Finally, the suspension of RBCs was added to 11 mL of PBS to 3 mL of red blood cells. The concentrations of the AC solutions were 6.125 nM, 12.5 nM, 25 nM, 50 nM, 100 nM, 1 μM, and 10 μM. These solutions were added to 1 mL of erythrocyte suspension and incubated for 4 h, 37 °C, followed by high-speed centrifugation at 20,000 *×* *g* for 15 min. The supernatant obtained were assayed for absorbance, absorbance (A) was measured at 570 nm using an enzyme marker. In the control group, the erythrocyte suspension was incubated in 1 mL of PBS (negative control) and double-distilled water (positive control).

### In vitro nitric oxide testing

After administration of AC, blood was taken from the mice orbit at 0 min, 5 min, 15 min, 30 min, 60 min, 90 min, 120 min, and 240 min in 1.5 mL tube containing sodium heparin. The supernatant was collected. The supernatant was used to detect the NO concentration. The NO content was measured with the procedure of the NO assay kit. The absorbance of the reaction was measured at 540 nm using a 96-well plate (Thermo Scientific).

### Statistical analysis

All statistical analyses were conducted using SPSS 20.0. One-way analysis of variance (ANOVA) tests with Tukey’s multiple comparisons tests and *t*-test were used to calculate adjusted *P* values (*n* ≥ 3, Mean ± SD). *P-*value of 0.05 or lower was considered statistically significant.

## Results

### Highly individualized AC-triggered cardiotoxicities were observed in DO mice

**Fig. 1:**
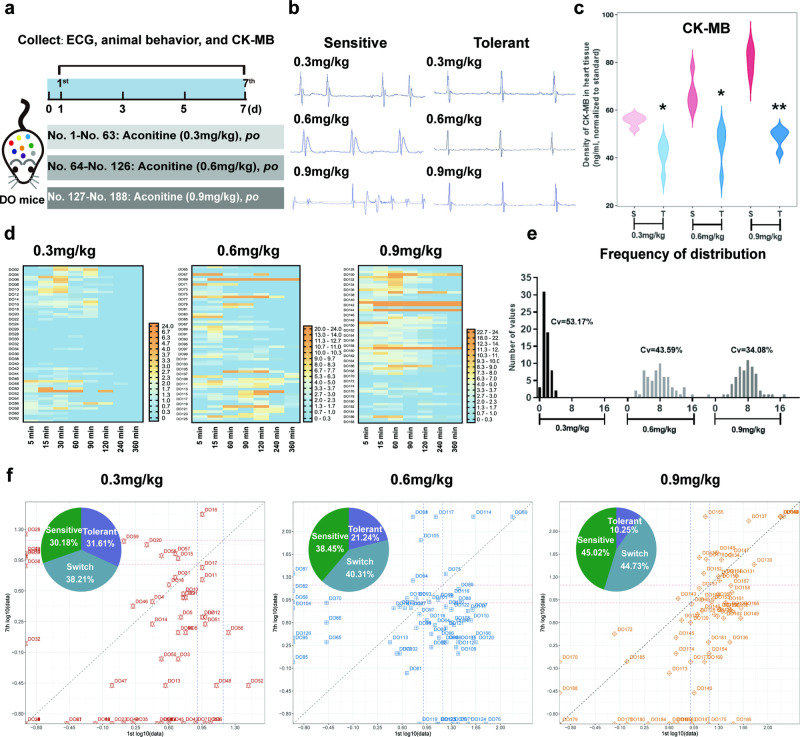
In DO mice, AC-triggered cardiotoxicity shared similar characteristics with clinical patients, and significant individual variabilities were also observed. **a** The schematic diagram showed DO mice grouping and the dosages of AC administrations. ECGs (**b**) and the levels of CK-MB (**c**) in AC-sensitive and AC-tolerant mice were measured after treatments of AC (*n* = 5). Cardiotoxicity-related behavior scores (**d**) and the coefficient of dispersion (**e**) of DO mice were recorded on the d 1. **f** The proportion of sensitive and tolerant mice at three dosages of AC. (*P < 0.05, **P < 0.01).

To comprehensively simulate the individual differences in AC-associated cardiotoxicities, genetically heterogeneous DO mice, each of which is analogous to the individual patient, were used. After exposure to AC (0.3 mg/kg, 0.6 mg/kg, and 0.9 mg/kg), DO mice with abnormal ECG, increased levels of CK-MB, a specific biomarker for cardiotoxicity, and behavior scores higher than 15 were defined as mice sensitive to AC-related cardiotoxicity, while those with the opposite responses were recognized as the AC toxicity-tolerant phenotype (Fig. 1a). Compared to ECG in AC-tolerant mice, AC-sensitive mice developed diverse types of arrhythmias, such as paroxysmal supraventricular tachycardia, atrial fibrillation, and fatal ventricular fibrillation (Fig. 1b). Moreover, the levels of CK-MB were markedly increased in AC-sensitive mice, and the difference between sensitive and tolerant mice increased in a dose-dependent manner (Fig. 1c).

Most interestingly, according to cardiotoxicity-related behavior scores, we found tremendous individualization in AC-exposed DO mice. ~17.40% of mice showed toxic symptoms when given 0.3 mg ·kg^−1^ ·d^−1^ AC, while that incidence increased to 55.56% and 73.01% after treatment with AC at 0.6 mg· kg^−1^ ·d^−1^ and 0.9 mg· kg^−1^ ·d^−1^, respectively (Fig. 1d). Correspondingly, the coefficient of dispersion was 53.17%, 43.59%, and 34.08% in 0.3 mg/kg, 0.6 mg/kg, and 0.9 mg/kg, respectively, indicating that in regard to a lower dosage, the individualized diversity of AC-induced cardiotoxicity would be more significant (Fig. 1e). Moreover, by combining data collected on the d 1 (initial exposure) and d 7 (terminal exposure) of AC treatments (Fig. S1), we found that ~30.18%–45.02% of DO mice were persistently sensitive to AC, while ~10.25%–31.61% of mice consistently remained tolerant (Fig. 1f). These findings suggest that the responsive sensitivity of DO mice to AC-triggered cardiotoxicity may predominantly depend on individual heterogeneity rather than treatment duration.

### Differential genetic background is the key determinant for the individualized response to AC-induced cardiotoxicity

**Fig. 2:**
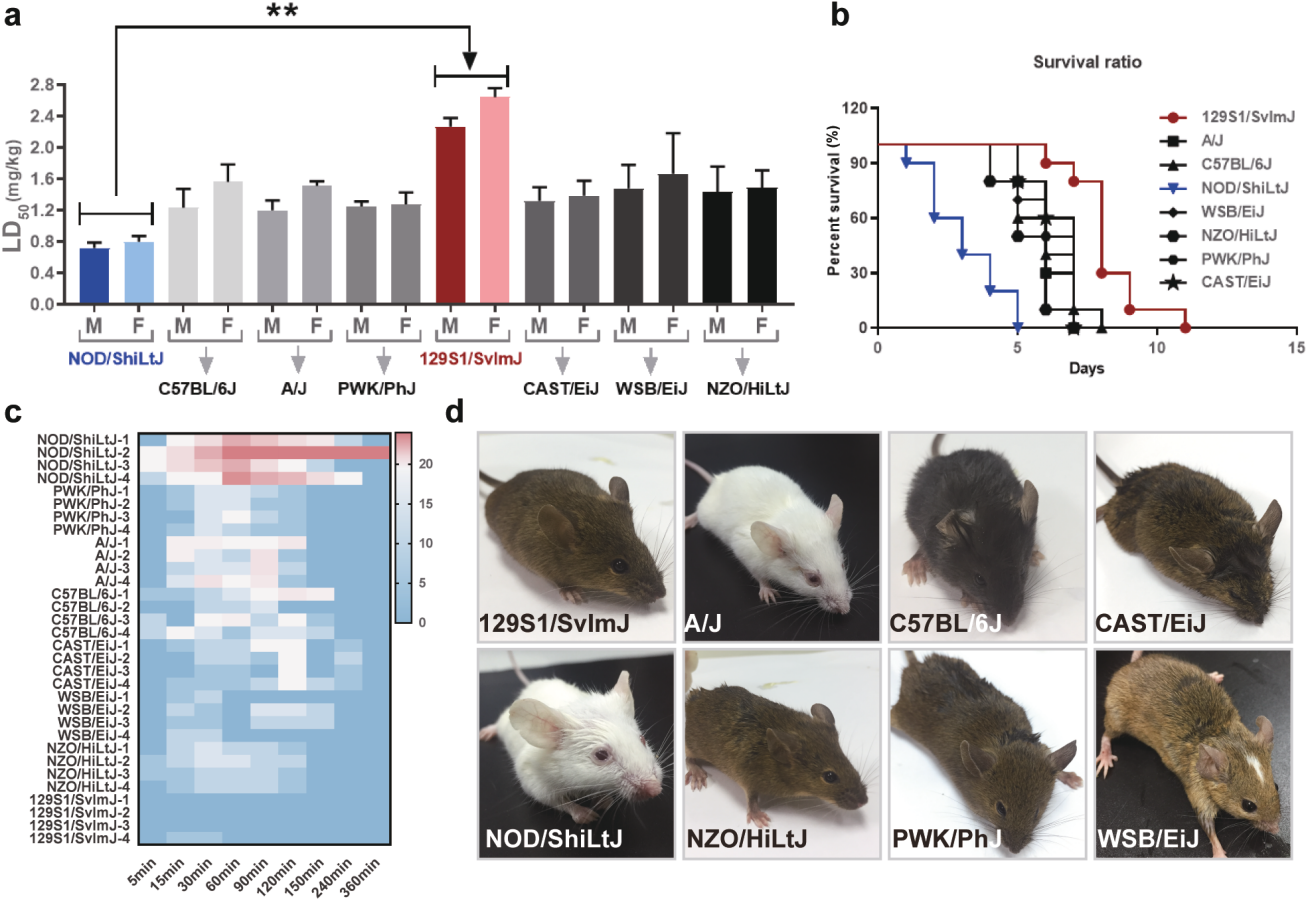
Differential gene background would be the key determinant for the individualized response of AC cardiotoxicity. **a** The LD_50_ of AC were measured in 8 progenitor mice of DO mice, either in female or in male mice, respectively (*n* = 10). **b** Survival ratio of 8 progenitor mice was measured (*n* = 10). **c** The behavior scores of progenitor mice were recorded within 0–360 min after exposed to AC at 0.405 mg/kg (1/3 of average LD_50_) (*n* = 4). **d** Representative images of progenitor mice after AC exposure. NOD/ShiLtJ mice suffered from vomiting, tachyarrhythmia, desudation, diarrhea, and convulsion, while in 129S1/SvImJ, no significant physical changes were observed. (**P < 0.01).

To determine the inherited genetic background of AC-sensitive and AC-tolerant mice in DO mice, the eight inbred progenitor mouse strains were subjected to screening. As shown in Fig. 2a, when exposed to AC, ~6 out of 8 progenitor moues strains shared similar LD_50_ values (~1.2 mg/kg −1.6 mg/kg). Among them, NOD/ShiLtJ was the most AC-sensitive strain, with the lowest LD_50_ of 0.723 mg/kg in males and 0.817 mg/kg in females, while 129S1/SvImJ mice were the AC-tolerant strain, with the highest LD_50_ of 2.141 mg/kg in males and 2.663 mg/kg in females. In addition, the survival ratio showed that when treated with AC at 1.215 mg· kg^−1^ ·d^−1^ (average LD_50_ dosage in 8 inbred mice), all NOD/ShiLtJ mice died within 5 d, while 129S1/SvImJ mice remained stable for 10–12 d (Fig. 2b). Furthermore, behavior scores also indicated that almost all NOD/ShiLtJ mice suffered from vomiting, tachyarrhythmia, desudation, diarrhea, and convulsion, while in 129S1/SvImJ mice, no significant physical changes were observed either before or after AC treatments (Fig. 2c, d).

Additionally, to further determine whether the concentration of AC or the differential genetic background in NOD/ShiLtJ and 129S1/SvImJ contributed more to their different responses toward AC-triggered cardiotoxicity, the concentration and tissue distribution of AC in these two mouse strains were evaluated. We found that the plasma concentrations or tissue distributions of AC, as well as its metabolites BAC and aconine, were not significantly different between AC-sensitive and AC-tolerant mice (Fig. S2). This evidence implied that hereditary genetic characteristics, rather than the elimination of toxic AC, may be the predominant factor determining individualized responses to AC-related cardiotoxicity.

### HBB is the master gene governing the responsive sensitivity in AC-induced cardiotoxicity

**Fig. 3:**
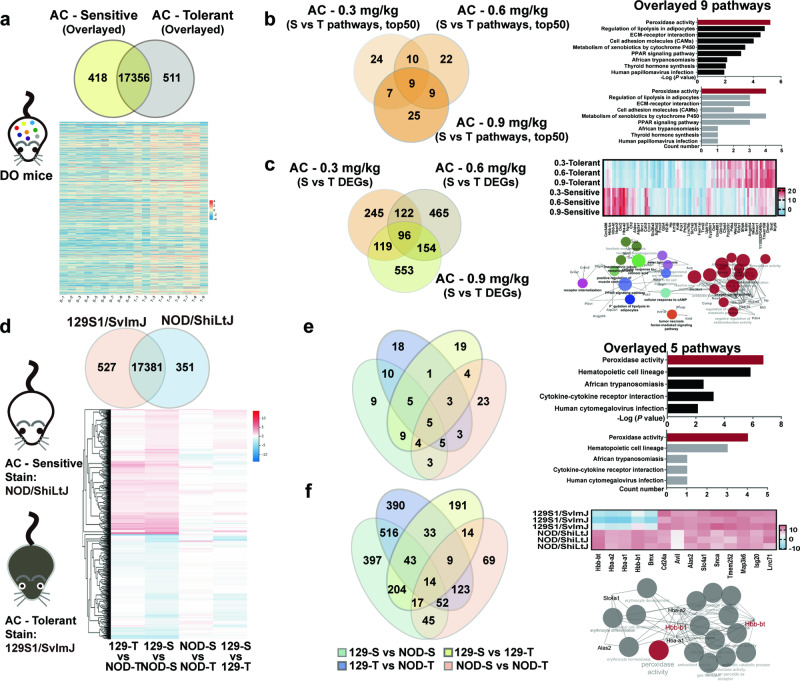
HBB is the only specifically expressed gene in AC-sensitive mice, overlayed in DO mice and progenitor mice. **a**–**c** Show the results in DO mice(*n* = 9). **d**–**f** Show the results in progenitor mice (*n* = 5). **a** The Overlapping DEGs (Venn diagram) and DEGs (Heat map). **b** Nine signaling pathways were overlayed (Histogram) and among top 50 signaling pathways enriched (Venn diagram). **c** 96 of DEGs were overlayed (Venn diagram), and DEGs expression were validated (Heat maps). Then the interaction was analyzed (network diagram)**. d** Overlapping DEGs (Venn diagram) and cluster analysis of DEGs (Heat maps). **e** The signaling pathways enriched (venn diagram) were overlayed and top of 5 overlayed pathways (histogram). **f** Overlapping DEGs (Venn diagram) and 14 overlayed DEGs expression were validated (Heat maps). KEGG and the interaction of 14 overlayed DEGs (network diagram) (fold change > 2).

To further identify specific biomarkers for individual differences in AC-triggered cardiotoxicity, RNA-seq was performed for either DO mice or mice of their progenitor strains (NOD/ShiLtJ as the AC-sensitive strain; 129S1/SvImJ as the AC-tolerant strain). For DO mice, 418 and 511 differentially expressed gene (DEGs) were specifically found in AC-sensitive and AC-tolerant mice, respectively (Fig. 3a). By analyzing the top 50 differentially enriched signaling pathways and DEGs detected in the 3 dosages of AC treatment, nine signaling pathways, and 96 DEGs were significantly enriched; among them, peroxidase activity was the most significant pathway, with the lowest *P* value (*P* = e^−5.76^) and highest count number (*n* = 4) (Fig. 3b, c). Seventeen DEGs, such as HBB, HBA, COMP, and NXPH3, were highly expressed in AC-sensitive mice, suggesting that these 17 genes could serve as specific cardiotoxicity-sensitive biomarkers (Fig. 3c).

For DO progenitor mice, by evaluating the individual responses of their offspring (F1), mice of the AC-tolerant 129S1/SvImJ strain were subclustered as absolutely tolerant (129-T) and relatively sensitive (129-S). Similarly, mice of the AC-sensitive NOD/ShiLtJ strain were also subclassified as absolutely sensitive (NOD-S) and relatively tolerant (NOD-T) (Fig. S3). A total of 527 and 351 DEGs were enriched in 129S1/SvImJ (absolutely tolerant) and NOD/ShiLtJ (absolutely sensitive) mice, respectively (Fig. 3d). By combining the signaling pathways enriched in the four comparisons, peroxidase activity remained the key pathway (Fig. 3e). Additionally, 3 DEGs, HBB, HBA, and BMX, were highly and specifically expressed in AC-sensitive mice (Fig. 3f). This evidence, from DO mice and confirmed in two DO progenitor strains, suggests that HBB could be the vital gene responsible for individualized AC-associated cardiotoxicity.

### HBB overexpression enhanced the responsive sensitivity of AC-induced cardiotoxicity

**Fig. 4:**
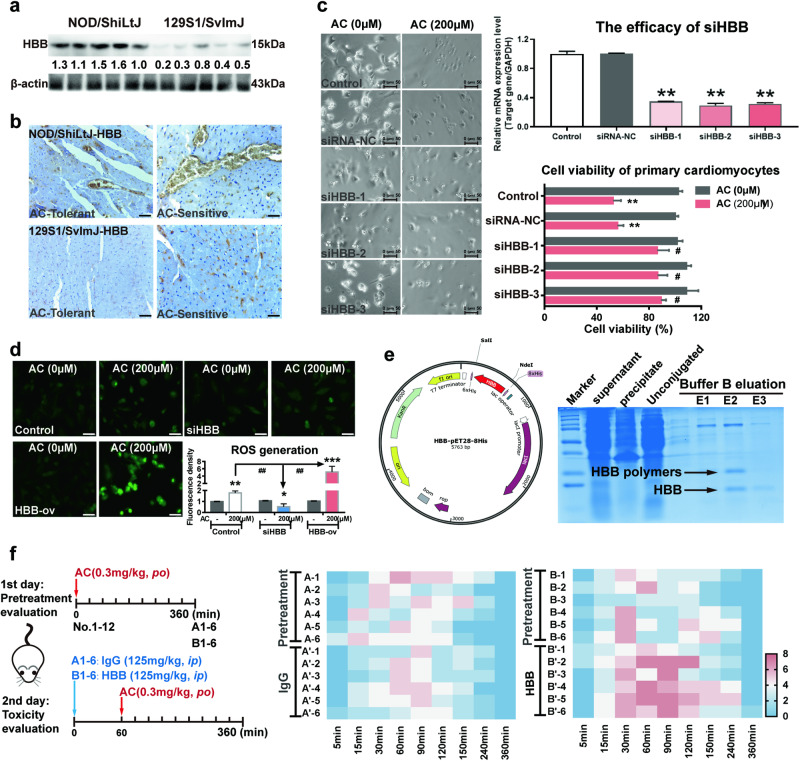
HBB overexpression enhanced the responsive sensitivity of AC-induced cardiotoxicity. **a**, **b** HBB expressions in heart tissues were evaluated by Western blotting and immunohistochemistry (IHC) (*n* = 5). **c** Cell viability assay of cardiomyocytes after exposed AC (*n* = 3). **d** The ROS level in primary-isolated cardiomyocytes was quantify(*n* = 3). **e** HBB protein expression and purification. **f** HBB pure proteins were given to mice. (HBB and IgG, 125 mg/kg, i.v, *n* = 6) (*P < 0.05, **P < 0.01, ***P < 0.001; ^#^*P* < 0.05, ^##^*P* < 0.01).

To determine the role of HBB in AC-induced cardiotoxicity, we initially examined HBB expression in the heart tissues of AC-sensitive mice (NOD/ShiLtJ) and AC-tolerant mice (129S1/SvImJ). As shown in Fig. 4a, HBB expression levels in 129S1/SvImJ mice were much lower than those in NOD/ShiLtJ mice. Furthermore, by comparing the IHC staining data of the offspring of NOD/ShiLtJ and 129S1/SvImJ mice, we also found that the order of increasing HBB expression levels was 129-T < 129-S < NOD-T < NOD-S (Fig. 4b). HBB expression knockdown in primary isolated cardiomyocytes resulted in the significant attenuation of AC-induced cell death (Fig. 4c). Since hemoglobin (Hb) functions to carry oxygen and scavenge ROS, intracellular ROS levels were detected after AC exposure. The ROS levels were significantly higher in AC-treated cells than in control cells, and in siHBB-treated cells, the AC-triggered ROS levels were dramatically decreased. In contrast, HBB overexpression enhanced ROS generation (Fig. 4d). To confirm the effect of HBB in vivo, HBB protein was purified (Fig. 4e) and administered to mice whose individual responses to AC had been normalized through the 1st round pre-evaluation (Fig. 4f). Compared to mice that received IgG, HBB-treated mice had significantly higher behavior scores related to AC-triggered cardiotoxicities (Fig. 4f). Moreover, to further confirm the key role of HBB in AC-related cardiotoxicity, the inhibitors of previously revealed AC-toxic targets were evaluated simultaneously. KB-R7943 and ranolazine, inhibitors of the well-known AC-toxic targets Na^+^/Ca^2+^ exchanger (NCX) and Na^+^/K^+^ exchanger, respectively, unexpectedly increased the toxic sensitivity and mortality by 42% and 75%. Furthermore, ranolazine significantly accelerated the death time from 30 min to 15 min (Fig. S4). All these results implied that HBB could markedly enhance AC-triggered cardiotoxicities as a specific and key player.

### AC could directly bind to HBB with the highest binding affinity

**Fig. 5:**
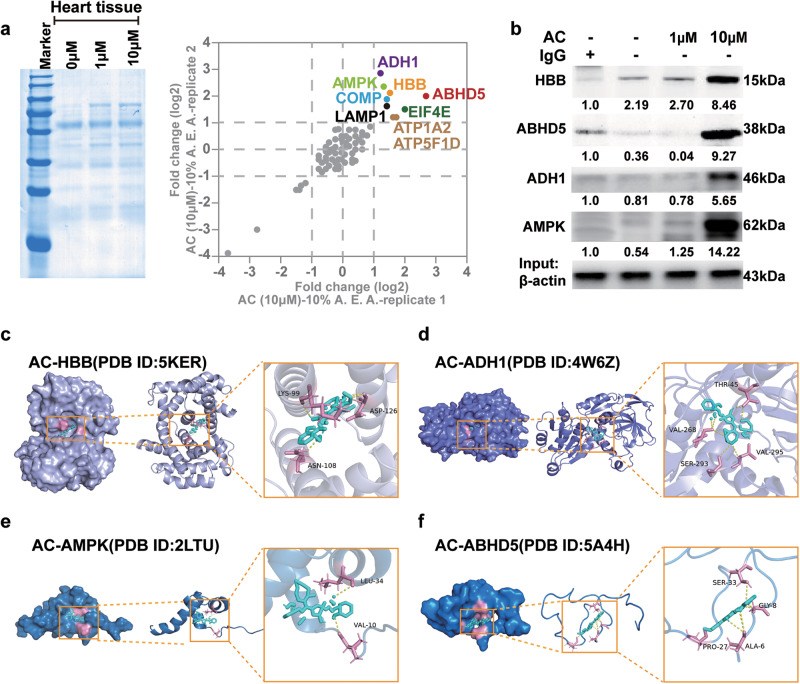
AC could directly bind to HBB with highest binding affinity. **a** The solvent-induced protein precipitation (SIP) combined with proteomics were utilized to identify the key targets for AC-associated cardiotoxicity. **b** CO-IP assay was applied to verify the combination of AC with HBB, ADH1, ABHD5, and AMPK. AC at 1 µM and 10 µM were used as concentration gradient to further confirm the bind of HBB with these proteins. **c**–**f** Docking data indicated the interactions of AC with HBB, ADH1, ABHD5, and AMPK, respectively and precise amino acids involved.

To identify the key targets for AC-associated cardiotoxicity, solvent-induced protein precipitation (SIP) was performed. As shown in Fig. 5a, the content of AC-conjugated proteins gradually increased with AC concentration (1 μM to 10 μM), and subsequently, LC‒MS/MS analysis revealed that HBB, ABHD5, ADH1, AMPK, COMP, EIF4E, LAMP1, ATP1A2, and ATP5F1D were the specific targets of AC. Furthermore, by combining CO-IP and docking data, we found that HBB, ABHD5, ADH1, and AMPK may directly bind to AC, and the binding efficiency increased with the concentration of AC (Fig. 5b). In particular, AC directly binds to HBB through the sites LYS-99, ASN-108, and ASP-126 (*K*_D_ = −15.7 kcal/mol) and binds to ADH1 through THR-45, VAL-268, SER-293, and VAL-295 (*K*_D_ = −17.73 kcal/mol). It binds to AMPK through VAL-10 and LEU-34 (*K*_D_ = −11.05 kcal/mol) and binds to ABHD5 through ALA-6, GLY-8, PRO-27, and SER-33 (*K*_D_ = −10.84 kcal/mol) (Fig. 5c–f). Moreover, we found that compared to other toxic compounds found in Fuzi, the binding affinity between AC and HBB was the highest (*K*_D_ = −15.7 kcal/mol). Furthermore, compared to many previously revealed targets of AC, the binding affinity of HBB and AC was still the highest (Fig. S5). These data suggest that among all AC targets, HBB would be the most predominant and promising target for AC-related cardiotoxicity.

### AC specifically binds to cell-free Hb to rapidly scavenge nitric oxide and induce cardiotoxicity

**Fig. 6:**
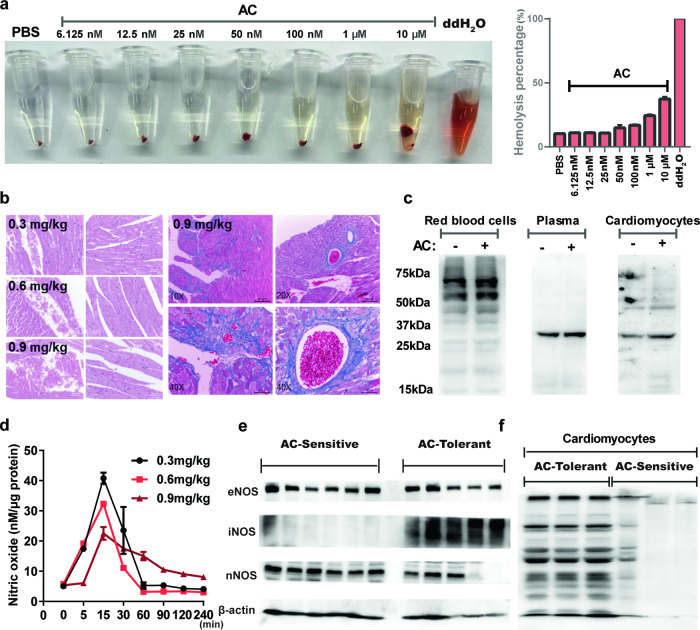
AC specifically binds to cell-free Hb to rapidly scavenge nitric oxide and induce cardiotoxicity. **a** The hemolysis effects of AC were evaluated from 6.125 nM to 10 μM in vitro. **b** HE staining of DO mice heart tissues in 0.3 mg/kg, 0.6 mg/kg, and 0.9 mg/kg group; as well as Masson staining in 0.9 mg/kg. **c** The interaction between AC and Hb in red blood cells, plasma, and cardiomyocytes. **d** NO generations in heart tissues were examined in DO mice within 0–240 min after treated with AC (*n* = 3). Protein expression levels of eNOS, iNOS, nNOS (**e**), and S-nitrosylation (**f**) were detected (*n* = 3).

To determine why HBB is dramatically overexpressed in AC-sensitive mice, the effect of AC on Hb dynamic transformation was studied. We found that AC markedly triggered hemolysis at concentrations from 100 nM to 10 μM (Fig. 6a). Simultaneously, accumulated red blood cells were also observed in the heart tissues of AC-sensitive DO mice in a dose-dependent manner, especially after AC (0.9 mg/kg) treatment, and mounds of red blood cells were congested around the tissues covered by collagen fiber (Fig. 6b). Since Hb is generally tetramerized and embedded in red blood cells, only when hemolysis occurs can cell-free hemoglobin (cf-Hb, Hb dimers) undergo decompartmentalization and be released into the plasma and surrounding tissues [16]. Consistently, we also found that in red blood cells, AC only binds to Hb tetramers (64 kDa), and in plasma and cardiomyocytes, AC preferentially binds to cf-Hb (32 kDa) (Fig. 6c). Given that the NO scavenging efficiency of cf-Hb is 6000 times greater than that of tetrameric Hb [17], we found that in the heart tissues of AC-treated DO mice, NO elimination was dose-dependently increased (Fig. 6d). Additionally, in AC-tolerant mice, damage-induced NO generation was more effective than that in AC-sensitive mice (Fig. 6e). Correspondingly, the levels of S-nitrosylation, the key protein posttranslational modifications maintaining Ca^2+^ homeostasis and heart rhythm during heart failure [18, 19], were also significantly decreased in AC-sensitive mice, implying that AC could bind to cf-Hb and effectively scavenge NO to induce cell death and fatal arrhythmia (Fig. 6f).

### HBB promotes AC-induced cardiotoxicity by interfering with the ABHD5/AMPK/HDAC4 axis

**Fig. 7:**
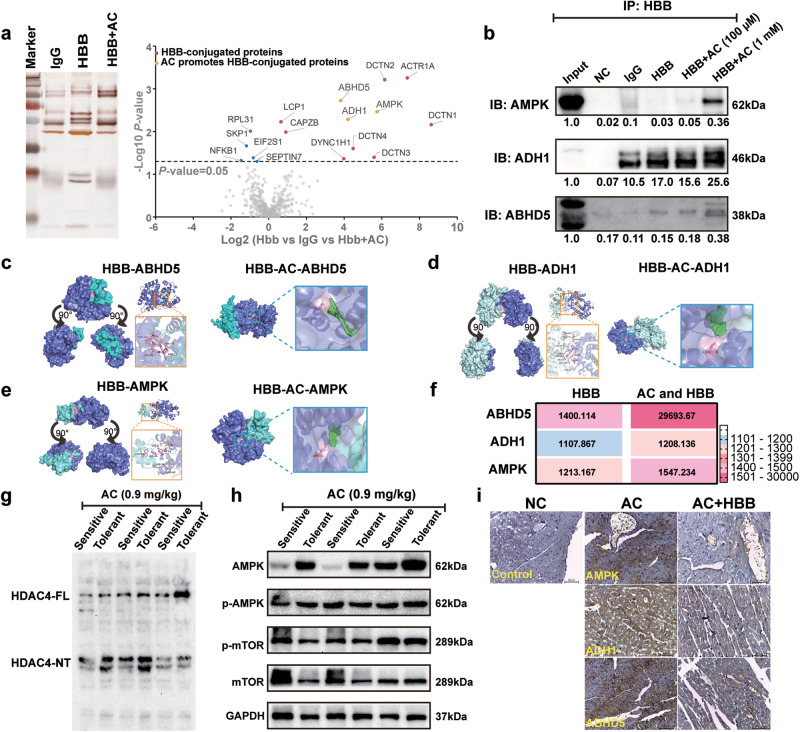
HBB promotes AC-induced cardiotoxicity via interfering with ABHD5/AMPK/HDAC4 axis. **a** The IP-mass assay was performed to determine HBB-binding proteins in HBB-overexpressed AC16 cells. **b** CO-IP assay was used to verify the binding of HBB with ADH1, ABHD5, and AMPK. Docking data (**c**–**e**) and the protein binding affinities (**f**) of HBB with ADH1, ABHD5, and AMPK before and after AC interfering. The protein levels of full length HDAC (HDAC-FL) (**g**), N-terminal polypeptide of HDAC4 (HDAC4-NT) (**g**), AMPK (**h**), p-AMPK (**h**), mTOR (**h**), and p-mTOR (**h**) were assessed (*n* = 3). **i** The protein levels of AMPK, ADH1, and ABHD5 were detected by IHC.

To further determine the relationship between HBB and other AC-specific targets, an IP-mass assay was performed in HBB-overexpressing AC16 cells. Silver staining revealed that compared to HBB alone, AC combined with HBB precipitated more proteins, especially increasing the binding ability of ABHD5, ADH1, and AMPK (yellow dots in Fig. 7a, b). Docking analysis also revealed that AC significantly promoted the interaction between HBB and AMPK and that between HBB and ABHD5 by increasing their binding affinities from 1213.167 kcal/mol to 1547.234 kcal/mol and from 1400.114 kcal/mol to 29693.67 kcal/mol, respectively (Fig. 7c–f). Previous studies demonstrated that ABHD5 could activate AMPK to further proteolyze HDAC4 into the N-terminal polypeptide of HDAC4 (HDAC4-NT) to protect against heart failure [19]. In agreement with previous studies, our data also revealed that in heart tissues of AC-sensitive DO mice, HDAC4-NT levels were significantly lower than those in AC-tolerant mice (Fig. 7g). Additionally, AMPK expression levels and subsequent mTOR and p-mTOR levels were also decreased dramatically in AC-sensitive mice (Fig. 7h). Moreover, when mice were treated with AC alone and AC combined with HBB, the basal expression levels of AMPK, ABHD5 and ADH1 were markedly decreased (Fig. 7i). These data suggest that AC may induce cardiotoxicity by promoting the interactions of HBB with ABHD5 and those of HBB with AMPK to synergistically inhibit the ABHD5/AMPK/HDAC4 axis.

## Discussion

Clinical evidence suggests that therapeutic agents such as Fuzi, warfarin, and digoxin have a narrow therapeutic index, and the patient response to drug efficacy or toxicity predominantly depend on the significant interindividual differences in each patient [20]. Research on personalized medicine, also known as individual or precise medicine, opened a new era in which therapeutic strategies transitioned from “one-size-fits-all” to tailored treatments based on individual characteristics, most of which were derived from genetic differences, environmental factors, and lifestyles. Previous studies have revealed that *Polygonum multiflorum*, a well-known herbal medicine that exerts high efficacy in relieving rheumatoid arthritis, is prone to cause approximately liver damage in 45.4% of patients who carry the HLA-B*35:01 allele [21]. Similarly, by utilizing the highly heterogeneous animal model of DO mice, each of which could be considered a mimic to each patient, we found significant individual differences in AC-induced cardiotoxicities. According to their diverse reactions in ECG, CK-MB, and physical behavior, some DO mice displayed a strong phenotype of tolerance to AC toxicity, while others showed sensitivity. The coefficients of variation were ~34.08%–53.17% (Fig. 1). To further investigate the genetic background of the AC-tolerant and AC-sensitive phenotypes, the eight progenitor strains of DO mice and their responses to AC toxicity were evaluated. Among them, NOD/ShiLtJ was recognized as an AC-sensitive strain, while 129S1/SvImJ was classified as an AC-tolerant strain (Fig. 2). By further determining the concentrations of AC in the sensitive and tolerant strains, we found that the toxicity-response differences were independent of its concentration and tissue distribution. These findings imply that AC-induced cardiotoxicity may be predominantly associated with AC toxicity-specific targets expressed in individuals (Fig. S2). RNA-sequencing of the heart tissues in DO, NOD/ShiLtJ, and 129S1/SvImJ mice indicated that hemoglobin subunit beta (HBB), a functional protein embedded in blood, was specifically enriched in AC-sensitive mice (Fig. 3). Most importantly, the amino acids of HBB in mice share ~89% similarity with those of HBB in humans. Thus, our findings provide evidence for HBB as a novel and promising toxic target for determining the individual differences in AC-induced cardiotoxicity, either in mice or in humans.

New mechanisms and toxic targets for AC-induced cardiotoxicity urgently need to be identified, and specific targets in the blood may provide evidence for the systematic explanation of the cardiotoxicity caused by AC. There are several reasons for this. *(i)* *Inhibitors of previously revealed AC toxicity targets could not significantly alleviate AC-triggered cardiotoxicity*. NCX is a well-known AC-toxic target, but its inhibitor only reversed ~28.6% of heart arrest cases [22]. More importantly, NCX inhibitors could only protect patients from heart failure after 8–24 weeks, while their effects on acute cardiotoxicity are small [23]. *(ii)* *New AC-toxic targets could be embedded in blood, which may exert key and direct effects in AC-induced cardiotoxicity*. Clinical observations revealed that neither cardioversion nor ~35.40% of antiarrhythmic therapeutics could successfully rescue AC-induced fatal ventricular arrhythmias [24]. In that case, promoting tissue oxygenation and charcoal hemoperfusion would be the ultimate and effective treatments for AC poisoning. This suggests that oxygen-saturated proteins in blood may directly interact with AC and determine the sensitivity of AC-induced cardiotoxicity [24]. Moreover, compared to ion channels, AC is more likely to selectively bind to fast-transforming and highly active proteins [7]. By evaluating the differential genes associated with AC toxicity sensitivity, either in high-heterogeneous DO mice (Fig. 3a–c) or in their progenitor mice (Fig. 3d–f), we found that HBB was the principal overlapping gene that was most highly attributed to AC-induced cardiotoxicity, and its overexpression could markedly increase cell death in cardiomyocytes by increasing ROS generation (Fig. 4). Most importantly, consistent with previous studies, our data also found that compared to the unexpected toxicity of KB-R7943 and ranolazine, which are inhibitors of the well-known toxic AC targets NCX and NKA (Fig. S4), supplementation with HBB significantly promoted AC-induced cardiotoxicity (Fig. 4f). These findings suggest that HBB could be the predominant protein that determines AC-related toxicity sensitivity.

Cell-free hemoglobin (Cf-Hb) is closely related to drug-induced myocardial injury and could serve as a promising predictive biomarker for determining AC toxicity sensitivity. Compared to N-terminal pro-B-type natriuretic peptide (NT-proBNP) and troponin T (troponin T), which are the classic predictive biomarkers for heart failure, cf-Hb in blood could be more sensitive in predicting heart damage in very early stages (OR, 1.31; 95% CI, 1.05–1.63; *P* = 0.02) [25]. Additionally, clinical trials have also revealed that cf-Hb is directly related to vascular damage and drug cardiotoxicity [26, 27]. In a recent study using multiomics sequencing to comprehensively explore the mechanism of cardiotoxicity caused by doxorubicin, it was found that the reduction in Hb oxygen-carrying capacity and the increase in DNA breakage are the two main pathways responsible for doxorubicin-induced cardiotoxicity, and the increased release of cf-Hb is a prerequisite for toxicity prior to DNA damage [28]. Consistently, we also found that AC could initiate the process of hemolysis (Fig. 6a), which subsequently induced hemoglobin (Hb) decompartmentalization and release into surrounding cardiomyocytes (Fig. 6b). Most interestingly, we found that AC could effectively bind to cf-Hb with higher affinity, especially in plasma and cardiomyocytes (Fig. 6c). Hb consists of two subunits, α and β, which form tetramers (64 kDa, 2α + 2β) embedded in red blood cells. When Hb is decompartmentalized and transformed to cf-Hb (32 kDa, 1α + 1β), it can easily be translocated into surrounding tissues. To further determine which subunit of cf-Hb, α or β, could directly bind to AC, chemical-protein actions were evaluated. We found that AC could directly bind to the β subunit of Hb at 15 kDa with a binding affinity of −15.7 kcal/mol (Fig. 5).

As a novel AC toxicity-specific biomarker, HBB may contribute to AC-induced cardiotoxicity in two ways. *(i)* *The first way is through interference with redox homeostasis*. A previous study showed that cf-Hb could participate in the redox homeostasis of cardiomyocytes by effectively scavenging NO and promoting ROS generation [29]. This could trigger myocardial local vasoconstrictive ischemia, exacerbate atherosclerotic plaque formation, and further aggravate cardiac injury and arrhythmia [30]. Recent studies have also demonstrated that NO can also affect myocardial remodeling by mediating S-nitrosylation of functional proteins in cardiomyocytes; thus, NO-mediated nitroso-redox imbalance has been shown to be the “fatal blow” leading to sudden cardiac death [31]. In our study, we found that AC could promote hemolysis, which facilitated the release of cf-Hb into surrounding cardiomyocytes to effectively scavenge NO and reduce S-nitrosylation (Fig. 6e, f). Moreover, HBB overexpression also significantly exacerbated ROS generation triggered by AC (Fig. 4d). *(ii)* *The second way is through interference with energy balance in the heart protective process*. To date, numerous cardioprotective targets in energy metabolism pathways have been identified. Among them, AMP-activated protein kinase (AMPK), an energy sensor, exerts significant cardioprotective effects by attenuating oxidative stress and cardiomyocyte apoptosis [32]. A previous study also revealed that ABHD5 could activate AMPK and subsequently decrease mTOR [33]. In our study, we found that AC could directly bind to HBB and promote the formation of tight binds between HBB and ABHD5 and AMPK (Fig. 7a–f), which resulted in low levels of active ABHD5 and AMPK in cardiomyocytes (Fig. 7i). While the expression levels of HBB were higher in AC-sensitive cardiomyocytes, ABHD5 and AMPK expression levels were specifically lower in AC-sensitive mice. Moreover, when AC was combined with HBB, ABHD5 and AMPK expression levels in heart tissues were significantly reduced (Fig. 7h, i). A previous study demonstrated that ABHD5 could further proteolyze HDAC4 into the N-terminal polypeptide of HDAC4 (HDAC4-NT) to protect against heart failure [34]. Consistently, we found that in AC-sensitive mice, which demonstrated significantly decreased levels of ABHD5, HDAC4-NT generation was markedly lower than that in AC-tolerant mice. These findings suggest that AC promoted the formation of HBB and that the ABHD5 complex significantly decreased HDAC4-NT levels, which exacerbated AC-induced cardiotoxicity.

In summary, our study found that significant individual differences were embedded in AC-induced cardiotoxicity, and HBB is a vital and novel toxic target for determining individualized responsive sensitivity via interference with the ABHD5/AMPK/HDAC4 axis. Our study not only provides the first evidence for determining the individual differences in AC-induced cardiotoxicity but also suggests a new strategy for exploring specific targets for the individualization of Fuzi treatment approaches.

### Supplementary information

Supplementary information

## Data Availability

RNA-seq (DO Mice: SUB10827138; 129S1-SvImJ mice: SUB10827093; NOD-ShiLtJ mice: SUB10827173) are available in Sequence Read Archive (SRA) database (Project No. PRJNA791298, PRJNA791308, and PRJNA791283, respectively). All the other data are available within the article and its supplementary materials.
