# Supplementary material for: HBB contributes to individualized aconitine-induced cardiotoxicity in mice via interfering with ABHD5/AMPK/HDAC4 axis
Source: Acta Pharmacol Sin. 2024 Mar 11;45(6):1224–36. doi: 10.1038/s41401-023-01206-3 (PMC11130212; doi:10.1038/s41401-023-01206-3)
Supplement: Supplementary file 1 — Supplementary information [file 41401_2023_1206_MOESM1_ESM.docx]

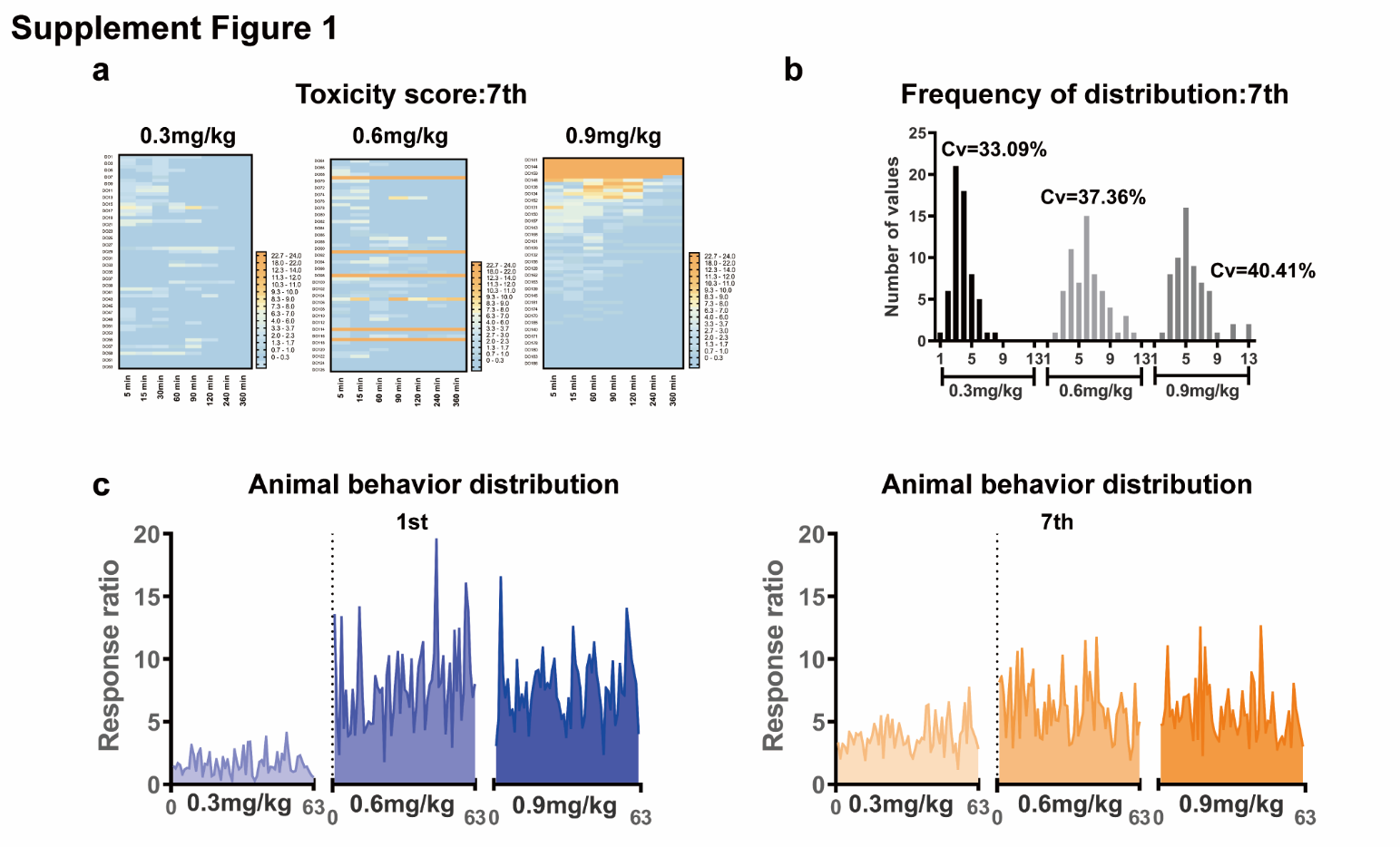


**Supplement Fig. 1. High-individualized AC-triggered cardiotoxicities were observed in DO mice. a** The cardiotoxicity-related behavior scores of AC-sensitive and AC-tolerant mice were measured on the d 7 (terminal exposure) after AC treatment at 0.3 mg·kg^-1^·d^-1^, 0.6 mg·kg^-1^·d^-1^, and 0.9 mg·kg^-1^·d^-1^ within 0-360 min. **b** The histogram of frequency distribution of behavior scores in DO mice after administrated with 0.3 mg·kg^-1^·d^-1^, 0.6 mg·kg^-1^·d^-1^, and 0.9 mg·kg^-1^·d^-1^ AC on the d 7. **c** The topographic map of frequency distribution of cardiotoxicity-related behavior scores in DO mice after exposed to 0.3 mg·kg^-1^·d^-1^, 0.6 mg·kg^-1^·d^-1^, and mg·kg^-1^·d^-1^ AC on the d 1 (initial exposure) and d 7 (terminal exposure) (0.3 mg·kg^-1^·d^-1^ group *n*=63, 0.6 mg·kg^-1^·d^-1^ group *n*=63, 0.9 mg·kg^-1^·d^-1^ group *n*=62).


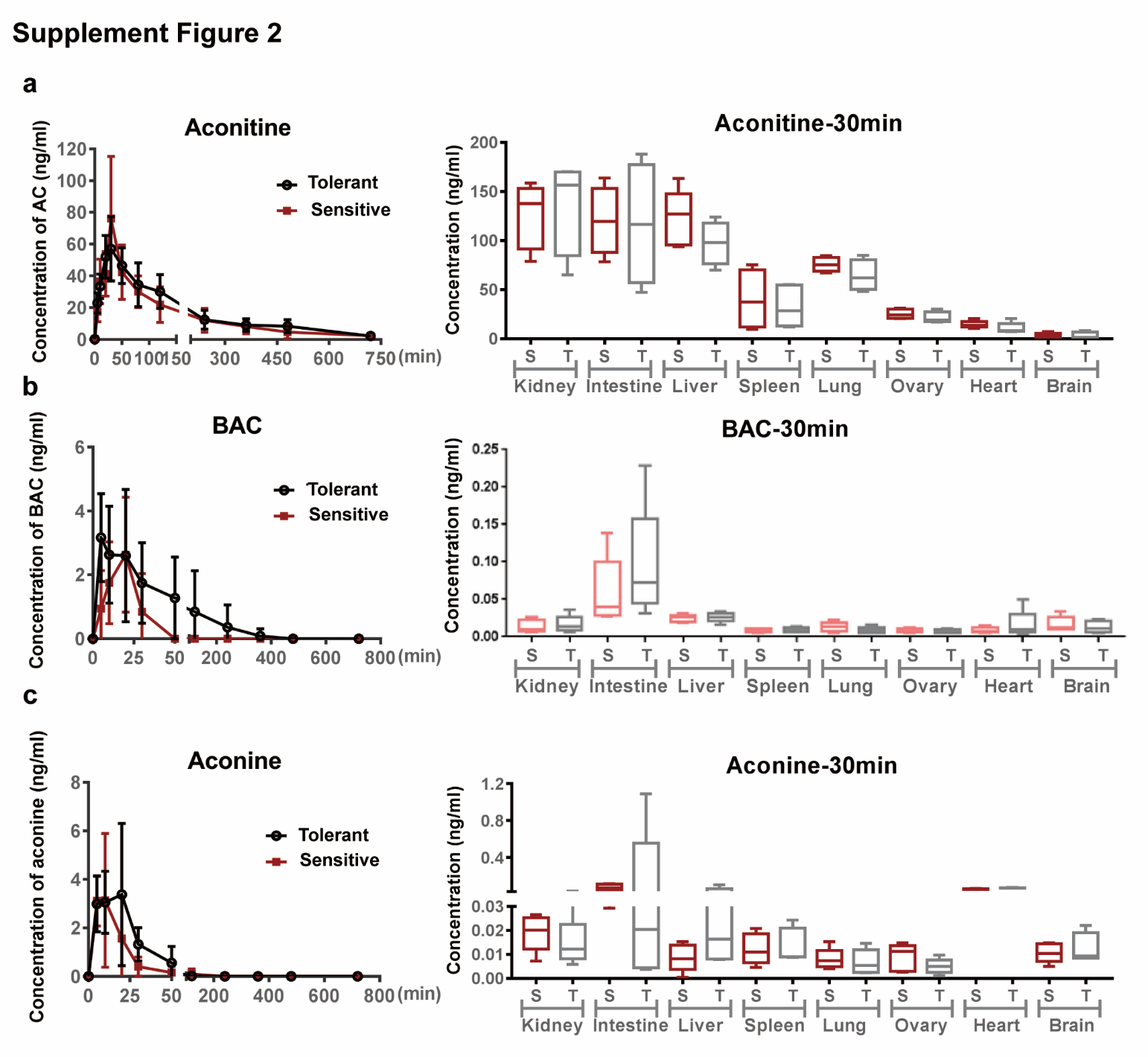


**Supplement Fig. 2** **Plasma concentration and tissue distribution of AC in NOD/ShiLtJ and 129S1/SvImJ mice. a-c** The concentration-time relationship of AC, BAC, and Aconine, as well as the tissue concentrations were measured by UHPLC-MS/MS. **Measurement of plasma concentration:** NOD/ShiLtJ and 129S1/SvImJ mice were gavaged 0.3 mg·kg^-1^·d^-1^ AC, for testing plasma concentration at 0 min, 5 min, 10 min, 20 min, 30 min, 50 min, 80 min, 120 min, 240 min, 360 min, 480 min, 720 min (*n*=25). **Measurement of tissue distribution:** NOD/ShiLtJ and 129S1/SvImJ mice, in half the number of females and half the number of males, 6-8 weeks, after given 0.3 mg·kg^-1^·d^-1^ AC for 30 min, kidney, intestine, liver, spleen, lung, ovary, heart and brain were taken, then which were used to examined tissue distribution (*n*=40).


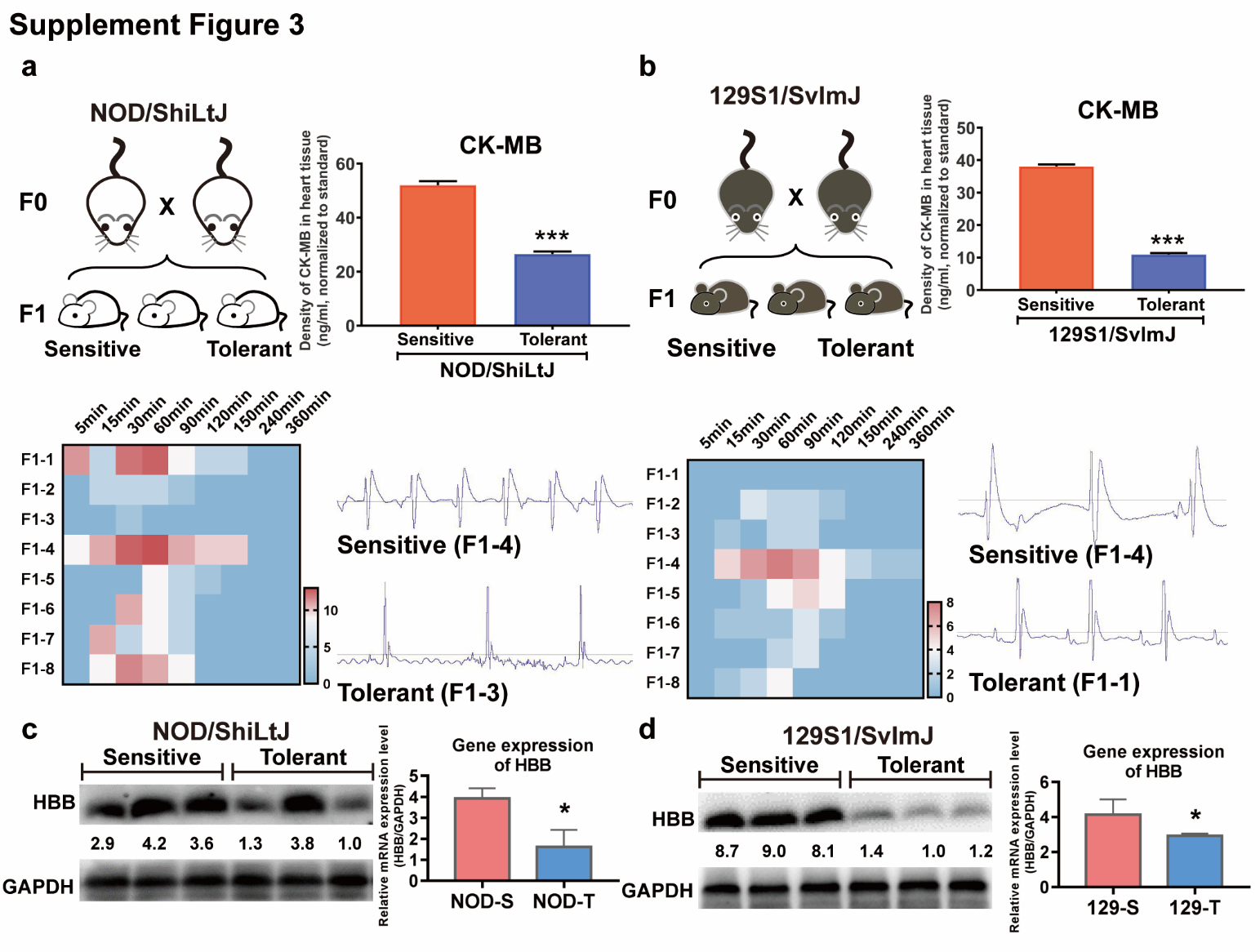


**Supplement Fig. 3 HBB is the master gene governed the switch of responsive sensitivity in AC-induced cardiotoxicity.** **a-b** CK-MB concentrations in the plasma of NOD/ShiLtJ and 129S1/SvImJ mice were assessed by ELISA kit (*n*=8). After administration of the indicated dose of AC, the cardiotoxicity-related behavior scores were recorded and ECGs were examined in AC-sensitive and AC-tolerant mice. Individual differences of AC toxicity existed in the offspring (F1) of the same strain of mice (NOD/ShiLtJ or 129S1/SvImJ). **c-d** HBB protein expression levels in heart tissue of absolutely tolerant (129-T) and relative sensitive (129-S), absolutely sensitive (NOD-S) and relative tolerant (NOD-T) mice were assessed by western blotting, as well as HBB gene expression levels were examined by RT-PCR (*n*=3). (****P* < 0.001, **P* < 0.05).


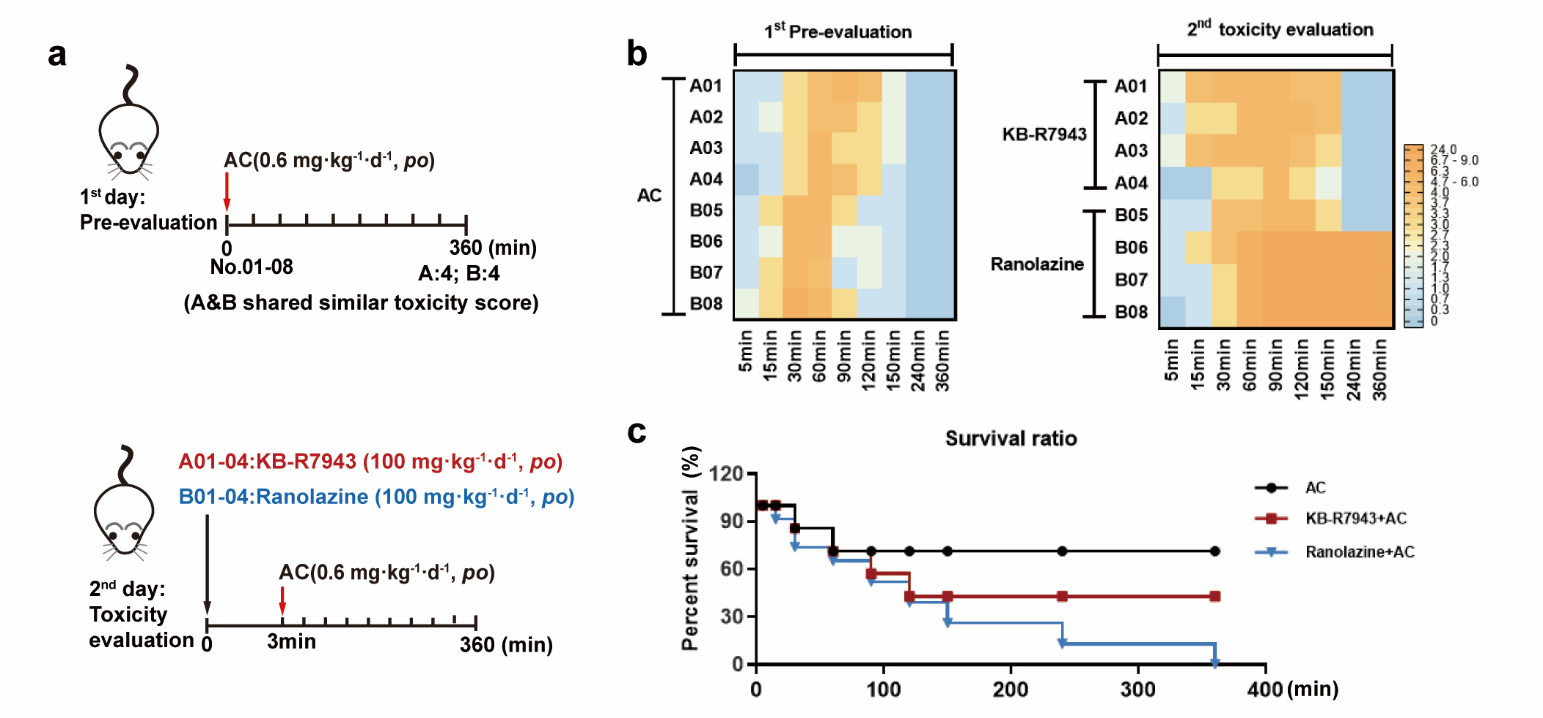


**Supplement Fig. 4 KB-R7943 and ranolazine could increase the AC toxic sensitivity and mortality.** **a** Timeline of AC treatment on the d 1. And Timeline of KB-R7943 and ranolazine respectively with AC treatment on the d 2. **b** Heat map of cardiotoxicity-related behavior scores on the d 1 and d 2. **c** The survival curves for 18 mice during 0-360 min after administration of AC, AC + KB-R7943 and AC+ Ranolazine, respectively (*n*=18).


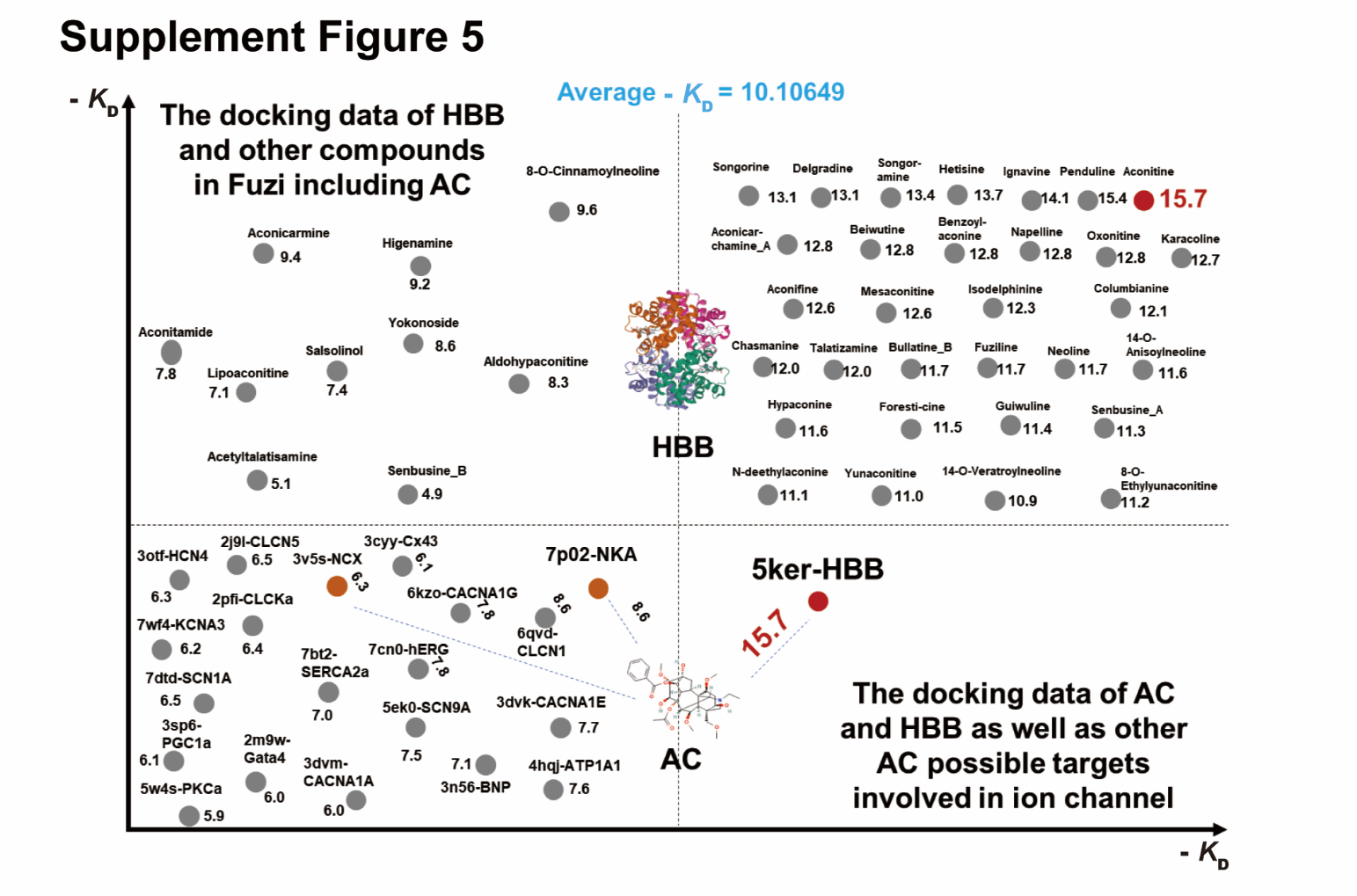


**Supplement Fig. 5** **Molecular docking of the major toxic components of Fuzi with HBB and AC with ion channel-like proteins.** The position of the dotted cross in the quadrant graph represents the negative value of the average binding ability: **10.10649 kcal**·**mol^-1^**. Above the dotted line on the parallel *X-axis* is the negative value of the binding ability of HBB with AC and its analogous compounds; below the dotted line on the parallel *X-axis* is the negative value of the binding energy of AC with ion channel proteins. The red dot in the figure marks the molecular docking binding energy of AC and HBB.
